# The effect of slow release insemination on pregnancy rates: report of two randomized controlled pilot studies and meta-analysis

**DOI:** 10.1007/s00404-017-4290-3

**Published:** 2017-02-14

**Authors:** Julian Marschalek, Maximilian Franz, Yael Gonen, Jan-Steffen Kruessel, Amnon Weichselbaum, Lorenz Kuessel, Marie-Louise Trofaier, Johannes Ott

**Affiliations:** 1 Department of Obstetrics and Gynecology, Medical University of Vienna, Waehringer Guertel 18-20, 1090 Vienna, Austria; 2 Lin Medical Center, Haifa, Israel; 3 Department of Obstetrics and Gynecology, Interdisciplinary Center for Reproductive Medicine (UniKiD), University of Düesseldorf, Düesseldorf, Germany; 4 Aqueduct Medical Ltd., Nazareth, Israel

**Keywords:** Slow release insemination, Intrauterine insemination, Pregnancy rate, Infertility, Outcome

## Abstract

**Purpose:**

A modified application technique of intrauterine insemination (IUI) is slow release insemination (SRI), first described by Muharib et al. (Hum Reprod 7(2):227–229, 1992), who postulated higher pregnancy rates with a slow release of spermatozoa for 3 h.

**Methods:**

To investigate this approach, two randomized controlled, cross-over pilot studies were performed from 2004 to 2006 in Israel and Germany to compare SRI with the standard bolus IUI. We aimed to present the results and perform a meta-analysis on available data for SRI. Univariate comparisons of pregnancy rates were performed using one-tailed *z* tests for method superiority. For meta-analysis, a fixed-effect Mantel–Haentzel weighted average of relative risk was performed.

**Results:**

Fifty treatment cycles (IUI: *n* = 25, SRI: *n* = 25) were performed in Germany, achieving four pregnancies (IUI: 4%, SRI: 12%, *p* > 0.05). Thirty-nine treatment cycles (IUI: *n* = 19, SRI: *n* = 20) were performed in Israel achieving six pregnancies (IUI: 10.5%, SRI: 20%; *p* > 0.05). Meta-analysis of all eligible studies for SRI (*n* = 3) revealed a combined relative risk for pregnancy after SRI of 2.64 (95% CI 1.04–6.74), *p* = 0.02).

**Conclusions:**

In conclusion, these results lend support to the hypothesis that the pregnancy rate might be improved by SRI compared to the standard bolus technique.

## Introduction

Intrauterine insemination (IUI) is a commonly used infertility treatment option for couples with unexplained subfertility, low-grade endometriosis, impotentia coeundi and sexual function disorders, as well as male subfertility [1]. The probability of conceiving with this infertility treatment depends on several factors, including age, reason for subfertility or infertility, absence/presence and type of ovarian stimulation, as well as the timing of insemination [2]. According to a large retrospective cohort analysis from the Netherlands, covering more than 15,000 IUI-cycles, the mean pregnancy rate was 5.6% per cycle. Cumulative ongoing pregnancy rates after the third, the seventh and ninth cycles were 18, 30, and 41%, respectively [3].

Many studies on IUI have dealt with strategies for improvement of pregnancy rates: the attention has focused on andrological factors, modification of controlled ovarian hyperstimulation (COH) protocols, and technique and timing of the IUI [2, 4, 5]. According to a recent Cochrane Database review, IUI with COH increases the live birth rate compared to IUI alone. The probability of pregnancy was also increased for IUI treatment in comparison with timed intercourse in stimulated cycles [6]. Usually, IUI is performed around ovulation and, as the fertile timeframe is limited, the correct timing of the insemination seems to be important. However, the quality of evidence to determine the potential difference in effectiveness between different methods of synchronization of ovulation and insemination is low [1, 4, 5].

Only a few studies have dealt with the technique of IUI itself or questioned the application method. Intratubal insemination (ITI) involves injection of washed sperm into the fallopian tube, although this procedure is no longer generally regarded as having any beneficial effect compared with IUI [7]. Another approach to the IUI technique was fallopian tube sperm perfusion (FSP) in the 1990s, mainly utilized in patients without tubal subfertility. It has been postulated that the presence of higher sperm densities in the fallopian tubes at the time of ovulation would be beneficial in comparison with IUI. However, available evidence suggests that there is no clear benefit for FSP over IUI [8]. Additional approach to the IUI method challenged the effect of timing of an IUI on the pregnancy success rate by performing two inseminations in a cycle [9].

Another modified application technique of IUI is the slow release insemination (SRI). Muharib et al. [10] postulated higher pregnancy rates than the standard bolus IUI with a slow release of spermatozoa utilizing a Grasby type MS16 pump for 3 h. In this randomized cross-over study, per cycle and cumulative pregnancy rates after four cycles were 6.1 and 22% for the standard IUI, and 15.0 and 63.1% for SRI, respectively [10]. The authors hypothesized that a persistent low concentration of spermatozoa might prolong the period of potential fertilization and thereby mimic physiological sperm transportation into the fallopian tube.

To investigate this new approach of SRI, two randomized controlled pilot studies were performed in Israel and Germany to compare the SRI with the standard bolus IUI treatment. We aimed to present the results of these studies, and in addition, we have performed a meta-analysis on available data on SRI.

## Materials and methods

### Study designs and patient population

We report on the results of two randomized controlled trials, conducted as pilot studies to compare SRI with the standard bolus IUI treatment. The first study was conducted at the Linn Medical Center, Haifa, Israel from November 2004 to June 2005, and entitled ‘Safety and efficacy of using slow release insemination method’. The second study was conducted at the Heinrich Heine University, Duesseldorf, Germany from January 2006 to June 2007, and entitled ‘Testing the effect of slow release of spermatozoa into the uterus on the pregnancy rate in women designated for artificial insemination’. Both studies were based on the same protocol that is presented in the following paragraphs. The studies were approved by the local ethics committees (Israeli ministry of health files, approval number: HTA2497, and ethics committee of the medical faculty of the Heinrich Heine University, registration number: HHU2705) in accordance with the Declaration of Helsinki. In the following text, the studies will be addressed as the “LIN study” and the “HHU study”, respectively.

Women were included if they fulfilled the following criteria: (1) primary or secondary infertility after 6 months of unprotected sexual intercourse; (2) age 20 to 40 years; (3) tubal patency as diagnosed by hysterosalpingography or chromopertubation with a maximum time interval between hysterosalpingography or chromopertubation and the woman’s enrollment into the study of 12 months; and (4) women with infertility due to anovulation and/or male factor with a minimum of >10 million of motile sperm cells per sample and/or endometriosis and/or unexplained infertility defined as the absence of a definable reason for a couple’s failure to conceive after 12 months of attempting conception despite a detailed evaluation of ovulation, tubal, and uterine as well as male factors. Patients with uterine abnormalities, including a septate uterus, presence of leiomyomas, or tubal subfertility, were excluded.

Both studies were conducted as randomized cross-over trials. The cross-over design was chosen, since it has been shown to lead to results comparable with studies with a parallel design, and thus to be a valid approach for infertility trials [11, 12]. Allocation to the first treatment was made at random by choosing an envelope containing the insemination type and women were randomized to one of the two following groups for the first treatment: (1) those undergoing the standard bolus IUI treatment and (2) those to be treated with the SRI method. Women who failed to conceive in the course of the first treatment were then offered the alternative method.

The primary outcome parameter was serological pregnancy defined as a positive beta HCG test (in urinary or blood samples) 2 weeks after insemination. In addition, the following patient and treatment-related parameters were reported: female factors: age, gravidity, cause of infertility, previous IUI treatment, reproductive medications, concomitant diseases; male factors: age, sperm motility, sperm count, and normal/abnormal sperm-percentage according to the analysis on the day of SRI/IUI.

### SRI technique

**Fig. 1:**
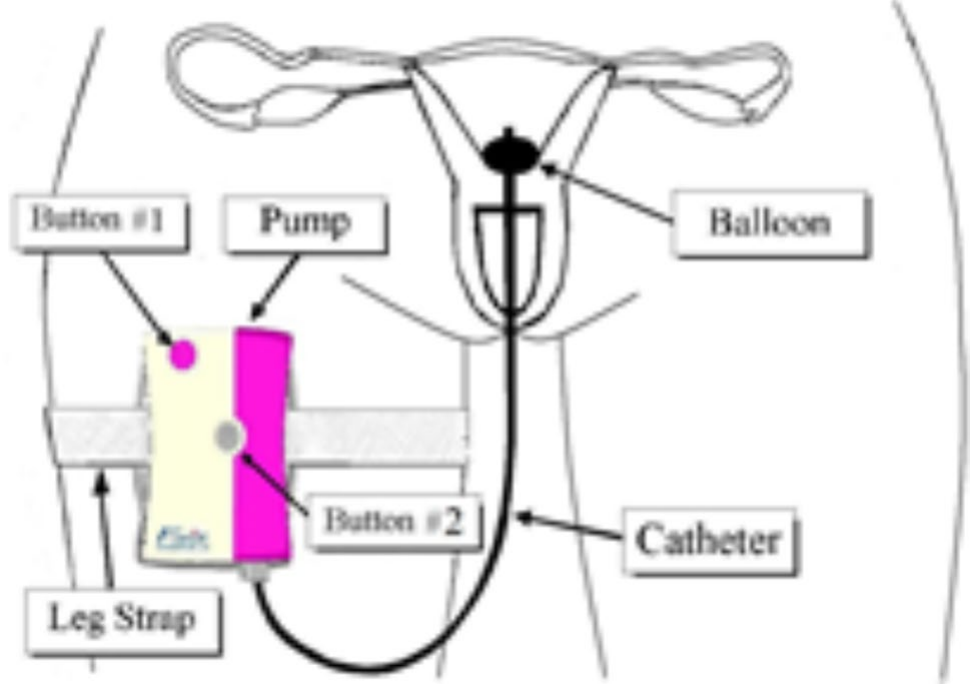
Schematic illustration of IQI-100 syringe pump

The Fertiligent device is composed of an ambulatory, disposable IQI-100 syringe pump (Fertiligent, Ra’anana 4325623, Israel), a BD 3 cc sterile syringe and a Cook HSG catheter with inflatable anchor balloon at the tip. The disposable pump (Fig. 1) is a mechanical spring driven device, where a spring pushes the syringe plunger with a patented spring restriction mechanism, which keeps it running during a 4-h period. The prepared sperm solution, located in a sterile sperm compatible chamber in the syringe, is subsequently pumped through the catheter. The insemination catheter is rinsed with sperm washing medium before usage, in order not to dry out during the 4-h procedure.

The catheter is inserted into the uterus in the same way as insertion to perform a standard IUI. After positioning the catheter into the uterine cavity, it is anchored by inflating a balloon with normal saline solution (1 ml) that is attached to the end of the catheter. During insemination, the pump is strapped alongside a patient’s thigh, and activated by an operational button.

In contrast to the standard IUI procedure, due to the anchor balloon, there is no need to rest in a supine or Trendelenburg position for several minutes after the insemination procedure. During the 4 h insemination process, the patient is mobile (completely ambulatory). After a 4-h continuous slow release injection of spermatozoa, the catheter is removed from the uterine cavity by deflating the balloon and the entire device is discarded.

### Technique of standard bolus IUI

The standard bolus IUI is accomplished with a polyethylene insemination catheter (5 French, 28 cm). Before performing the IUI, the catheter is joined to a tuberculin syringe with a volume of 1 ml.

The insemination syringe contains laboratory prepared sperm and is connected to the insemination catheter, which is inserted into the uterine cavity. After sperm injection and catheter removal, the woman normally rests in supine or reverse Trendelenburg position for 10 min.

### Meta-analysis

#### Data collection

Medline, EMBASE, and the Cochrane controlled trial register were searched to identify controlled trials, cohort studies, systematic reviews, and meta-analyses evaluating fertility outcome after slow release insemination (search date: April 18th 2015; search terms: intrauterine insemination; slow release insemination). Studies were included if they were published as complete reports in English. Bibliographies of studies were searched for relevant citations. According to the protocol, multiple studies describing the same study population would be included only once using the original publication, i.e., the one with the earliest date of publication. All eligible studies had to have the exact number of the patients, the exact rates of pregnancy after slow release insemination. Two authors assessed the eligibility of the studies and extracted relevant data (JM and JO). Missing information and additional trials were not sought from authors. Only one eligible study could be identified, published by Muharib et al. [10].

#### Risk of bias in individual studies

To ascertain the validity of the study, the methodological quality was analyzed on the basis of information reported in the original publication. By checking the adequacy of randomization, the completeness of follow-up and outcome reporting the study design was tested.

## Statistical analysis

For numerical parameters, variables are summarized as either mean ± standard deviation (SD) or median with interquartile range (IQR) depending on the data distribution. For categorical parameters, data are presented as frequencies and percentages.

For analyses of the LIN and the HHU studies, patients were divided into two groups: those who underwent IUI versus those who underwent SRI. Chi-squared Fishers’ Exact tests were used to compare nominal variables between the two groups and independent-samples median tests for numerical variables. One-tailed *z* tests were used to test for superiority of the pregnancy rate of SRI over IUI (RR > 1).

All treatment comparisons used the IUI group as the reference (comparator).

*p* values, relative risk (RR) with 95% confidence intervals (95% CI), are reported. IBM SPSS 22.0 (SPSS 22.0, SPSS Inc., Chicago, IL, USA) was used for statistical analysis.

For meta-analysis, a fixed-effect Mantel–Haentzel weighted average of relative risk was performed using RevMan 5.0 (RevMan 5.0, The Cochrane Collaboration, London, UK) to produce a combined relative risk (RR).

*p* values <0.05 were considered statistically significant.

## Results

### HHU study

**Fig. 2:**
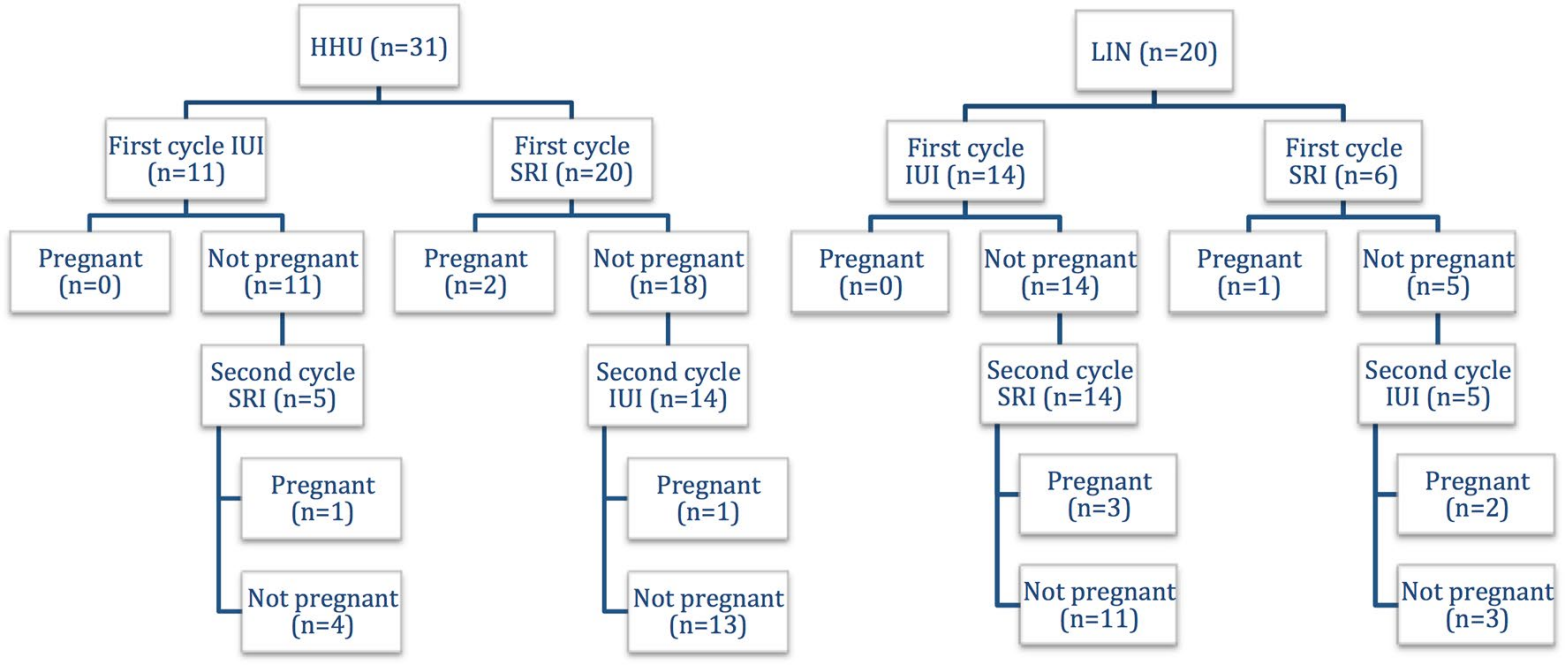
Randomization and pregnancy rate of HHU and LIN patients

A total of 31 women were randomized into the two-treatment arms: 11 (35.5%) women received a first standard IUI treatment and 20 (64.5%) women received a first SRI treatment. Nineteen women underwent a second treatment cycle (IUI: *n* = 14; SRI: *n* = 5). A study flow chart is provided in Fig. 2, and general population characteristics are presented in Table 1. There were no adverse events related to the use of SRI.

**Table 1:** Population characteristics of the HHU and the LIN studies (per case/treatment)

	HHU study (*n* = 50)	LIN study (*n* = 39)	*p* value
Female age (years)*	36.0 (34.5–38.0)	34.0 (27.0–36.0)	0.006
Previous pregnancies			0.125
0^#^	35 (70.0)	22 (56.4)	
1^#^	11 (22.0)	12 (30.8)	
>1^#^	1 (2.0)	5 (12.8)	
Missing data^#^	3 (6.0)	0 (0)	
Cause of infertility			<0.001
Endometriosis^#^	23 (46.0)	0 (0)	
Anovulation^#^	4 (8.0)	8 (20.5)	
Male factor^#^	7 (14.0)	0 (0)	
Unexplained infertility^#^	15 (30.0)	31 (79.5)	
Other^Ω^	1 (2.0)	0 (0)	
Male age (years)*	39.0 (36.0–41.0)	32.0 (31.0–35.5)	0.004
Semen analysis^†^			
Concentration (Mio/ml)*	50–59	20–29	0.025
Motility (%)^#^	40.0 (30.0–50.0)	33.0 (26.0–45.75)	0.034
Abnormal morphology (%)^#^	91.0 (85.0–94.0)	86.0 (80.0–89.0)	0.096

Data are presented as *median (interquartile range) or ^#^ *n* (%)

^Ω^Other cause of infertility—e.g., inhostile cervical mucous

^†^Semen analysis—after preparation, median values and IQR are provided for all IUI/SRI cycles; Semen concentration—data provided as median ranges

In total, there were 50 treatment cycles (IUI: *n* = 25, SRI: *n* = 25). There were no significant differences with respect to female and male population characteristics (Table 2). A total of four pregnancies were achieved following the different treatments: one (4.0%) in the IUI group and three (12%) in the SRI group. Assuming all treatments to be independent events, SRI had a relative risk of 3.000 (95% CI 0.3343 to 26.9202) compared to IUI, not reaching statistical significance (*z* = 0.981, *p*_(one−tailed)_ = 0.1632).

**Table 2:** Population characteristics of the IUI and SRI subgroups (per Insemination)

HHU (*n* = 50)	IUI (*n* = 25)	SRI (*n* = 25)	*p* value	LIN (*n* = 39)	IUI (*n* = 19)	SRI (*n* = 20)	*p* value
Female age (years)*	37.0 (35.0–38.0)	36.0 (32.0-38.5)	0.777		34 (27.0–36.0)	33.5 (27.0–36.0)	0.863
Previous pregnancies			0.803				0.916
0^#^	18 (72.0)	17 (68.0)			11 (57.9)	11 (55.0)	
1^#^	6 (24.0)	5 (20.0)			6 (31.6)	6 (30.0)	
>1^#^	0 (0.0)	1 (4.0)			2 (10.5)	3 (15.0)	
Missing data^#^	1 (4.0)	2 (8.0)			0 (0.0)	0 (0 0)	
Cause of infertility			0.711				0.935
Endometriosis^#^	10 (40.0)	13 (52.0)			0 (0.0)	0 (0.0)	
Anovulation^#^	2 (8.0)	2 (8.0)			4 (21.1)	4 (20.0)	
Male factor^#^	4 (16.0)	3 (12.0)			0 (0.0)	0 (0.0)	
Unexplained infertility^#^	9 (16.0)	6 (24.0)			15 (78.9)	16 (80.0)	
Other^Ω^	0 (0.0)	1 (4.0)			0 (0.0)	0 (0.0)	
Reproductive medication^‡^							
Clomifen citrate^#^	17 (68.0)	19 (76.0)	0.529		3 (15.8)	3 (15.0)	0.946
Gonadotropine therapy^#^	2 (8.0)	3 (12.0)	0.637		9 (47.4)	7 (35.0)	0.433
Ovulation induction^#^	16 (64.0)	15 (60.0)	0.771		17 (89.5)	19 (95.0)	0.517
Other^#^	3 (12.0)	5 (20.0)	0.440		1 (5.3)	3 (15.0)	0.316
Male age (years)*	39.0 (36.0-41.5)	37.0 (35.0–41.0)	> 0.999		32.0 (31.0–36.00)	32.0 (31.0–36.0)	> 0.999
Semen analysis ^†^							
Concentration (Mio/ml)*	60–69	40–49	0.067		30–39	20–29	0.991
Motility (%)*	50.0 (30.0–55.0)	40.0 (30.0–50.0)	0.256		33.0 (29.75–48.5)	34.0 (19.5-43.25)	> 0.999
Abnormal morphology (%)*	91.0 (85.0–94.0)	89.0 (84.5–94.0)	0.981		86.0 (79.5–89.0)	86.5 (79.5–89.0)	> 0.999
Treatment cycle			0.009				0.006
1^#^	11 (44.0)	20 (80.0)			14 (73.7)	6 (30.0)	
2^#^	14 (56.0)	5 (20.0)			5 (26.3)	14 (70.0)	

Data are presented as *median (interquartile range) or ^#^ *n* (%)

^Ω^Other cause of infertility—e.g., inhostile cervical mucous

^‡^Reproductive medication—data presented as cumulative reproductive medication, multiple entries possible

^†^Semen analysis—after preparation

### LIN study

Twenty women were randomized into the two-treatment arms: 14 (70.0%) women received a first standard IUI treatment and six (30.0%) women received a first SRI treatment. Nineteen women underwent a second treatment cycle (IUI: *n* = 5; SRI: *n* = 14). The according study flow chart is presented in Fig. 2 and Table 1 which gives detailed information on general population characteristics. There were no adverse events related to the use of SRI.

In total, there were 39 treatment cycles (IUI: *n* = 19, SRI: *n* = 20). Neither female nor male population characteristics differed between the IUI and the SRI groups (Table 2). A total of six pregnancies were achieved following the different treatments arms: two (10.5%) in the IUI group and four (20%) in the SRI group. Assuming all treatments to be independent events, SRI had a relative risk of 1.900 (95% CI 0.3925 to 9.1968) compared to IUI. This did not reach statistical significance (*z* = 0.798,* p*_(one−tailed)_ = 0.2125).

### Meta-analysis

#### Assessment of previously published studies

Literature review revealed only one previous study that compared SRI with the standard IUI technique [10]. This study followed 38 women, aged 24–36 years, in a cross-over study using up to four alternating cycles. The authors reported that “Thirteen pregnancies were achieved, nine after treatment A [slow release] (15.0%) and four after treatment B [bolus technique] (6.1%) (chi-squared = 2.7143, *p* < 0.05, using one-tailed probability)” [10]. According to this report, this would result in an uncorrected Chi square test statistic of 2.714 (with 1 *df*). Muharib et al. used a one-tailed test, and a significant *p* value of *p* < 0.05 was reported. However, this *p* value was calculated by halving the *p* value corresponding to the Chi-squared value obtained, with the intention of giving a *p* value for a one-sided test. This is incorrect, as a Chi-squared test compares distributions and is directionless.

We re-calculated these data using the *z* test which resulted in a relative risk (RR) of 2.475 for SRI compared to IUI (95% CI, 0.8037 to 7.6215). The *z* value for this RR is 1.579, which has an associated one-tailed *p* value of 0.057. In other words, the results reported by Muharib et al. do not reach statistical significance at the 5% level.

#### Meta-analysis of HHU/LIN study data and Muharib study

The meta-analysis shows that the combined relative risk of the three studies is 2.64 (95% CI 1.04–6.74) with a *p* value of 0.02, indicating a statistically significant advantage of SRI over conventional bolus IUI (Table 3).

**Table 3:**
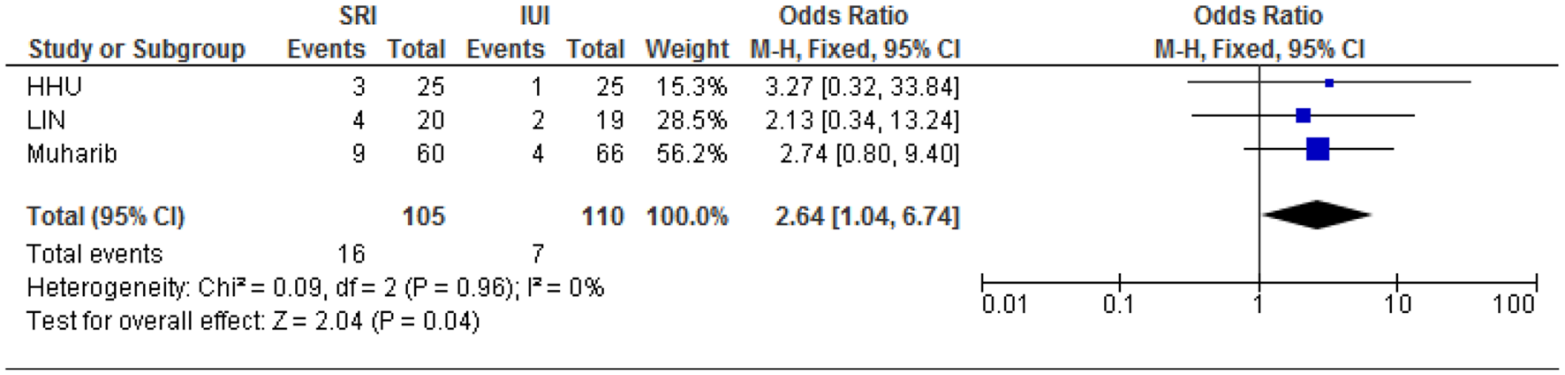
Results of combining relative risk (RR) from published and primary data sources

## Discussion

Both randomized controlled pilot studies show a trend towards higher pregnancy rates using the SRI method, even though the differences did not reach statistical significance. A meta-analysis, including these findings and the results previously published by Muharib et al., revealed a statistically significant advantage of SRI over conventional bolus IUI.

Muharib et al. [10] first described the new technique of SRI postulating that the pregnancy rate could be improved using this kind of insemination with a small and continuous number of spermatozoa released into the uterine cavity compared to the standard bolus technique. Although our re-analysis of their data indicates that this difference failed to reach statistical significance (*p* = 0.057), our meta-analysis confirms the superiority of SRI over IUI that had been initially assumed by Muharib et al. [10].

How can a better outcome after SRI be explained? Hypothetically speaking, the single deposition of a large number of spermatozoa into the uterine cavity during conventional IUI might cause polyspermia on one hand and discharge of spermatozoa through the fallopian tubes into the peritoneal fluid on the other hand [13, 14]. The concept of Fallopian tube sperm perfusion ensures the presence of even higher sperm densities in the fallopian tubes at the time of ovulation than does standard bolus IUI and has already been proven to have no clear benefit over IUI [8]. Therefore, it might be time to revise the theory that direct passage of highly concentrated spermatozoa through the uterine cavity and fallopian tubes will increase the density of capacitated spermatozoids near the oocyte and, therefore, subsequently, the chance for pregnancy [15].

Our data and those of Muharib et al. [10] support the concept that a small and continuous number of spermatozoa released into the uterine cavity might prolong the period of potential fertilization and thereby mimic physiological sperm transportation into the fallopian tube. Theoretically, the prolonged duration of insemination catheter-usage in SRI may stimulate local prostaglandin production and thereby might improve the transport of spermatozoa along the Fallopian tube.

It is well known that age has an important impact on the success rates after insemination and physiologically decrease over the course of a women’s life [16–18]. Cumulative pregnancy rates for both SRI and IUI were higher in the LIN study than in the HHU study, and notably, this was accompanied by significantly lower male and female age in the LIN study. Schorsch at al. reported that the pregnancy rate per patient was significantly higher for women below the age of 25 compared to women aged 35 years and over, claiming per-cycle pregnancy rates between 5 and 15% up until the sixth insemination [16]. Besides, there is evidence postulating an adverse effect of male age on clinical pregnancy rates, which is not only mediated by decreased sperm concentration or motility [19]. Of note, all patients participating in Muharib et al’s study were younger than 37 years. To what extent advanced male and female age influence pregnancy rates after SRI needs to be addressed in further studies.

One might argue that pregnancy rates after the standard IUI were low in the HHU study (4.0%) and in the study of Muharib et al. (6.1%) [10]. A high median female age and a high rate of endometriosis patients are notable in the HHU study (Table 1). Lower ovarian reserve, increased female age, as well as endometriosis have already been mentioned to negatively affect IUI outcome [20]. We cannot, however, comment on the pregnancy rate after IUI in the study by Muharib et al., since data were not sought from the authors. Neither did the authors provide exact data on patient characteristics, nor did they comment on possible reasons for the poor outcome in their original report [10]. Notably, there were important differences between the three studies concerning ovarian stimulation. However, in the HHU and LIN studies, types of ovarian stimulation were distributed equally between the IUI and SRI groups (Table 2), whereas Muharib et al. had not used ovarian stimulation at all [10]. Although ovarian stimulation is well known to improve IUI outcome [2, 21], its lack in some cases should not have influenced the main results of this meta-analysis.

Since data were derived between 2004 and 2007, one could also criticize the delay in publication. We are aware of this issue. This is due to the fact that the interest in SRI had declined, but SRI had re-entered the focus of the device’s provider, namely, Fertiligent, within the last few years. Thus, data were provided to the authors in the beginning of 2016.

We are aware of the fact that the cross-over study design of the HHU and LIN studies might be seen as a major limitation. Cross-over studies with repeated interventions on the same women were not traditionally recommended for trials, where a successful outcome will have a permanent serial effect (pregnancy) that results in the withdrawal of the woman from the second arm of the trial. Pregnancy after the first treatment will, therefore, unbalance the research design and introduce a period effect. For this reason, some authors reject the utilization of this study design in infertility trials [22, 23]. However, this approach has been claimed to be an efficient and pragmatic design, particularly as only one cycle of each treatment is given to each woman. Cross-sectional studies have been shown to be a valid approach in infertility research [11]. In a recent study, Takada and colleagues reported that the cross-over design has the highest power and the smallest bias [12]. Hence, they recommended using a combination of cross-over design and Mantel–Haenszel method for two-period, two-treatment clinical trials with irreversible endpoints. Moreover, one might argue that both randomized controlled pilot studies included only a small number of women. This was due to a lack of funding. However, we consider it justifiable to include them into a meta-analysis, since literature lacks data on SRI. Another limitation is caused by inclusion of the Muharib study in the meta-analysis as study designs differed with respect to the number of performed cycles and the duration of SRI application.

To the best of our knowledge, this study is the first since 1992 evaluating this novel approach to intrauterine insemination: we were unable to find any other report that explored the SRI technique that had been described by Muharib et al. for the first time. We consider it a crucial strength of our report that we were able to combine all three data sets in a meta-analysis, although this combination has its limitations as discussed above.

In conclusion, these data lend support to the hypothesis that the pregnancy rate might be improved by using SRI rather than IUI. A randomized controlled multicenter trial in this area is currently underway using the successor to the IQI-100 system, known as EVIE.
